# C-reactive protein kinetics as a predictive marker for long-term outcome of immune checkpoint inhibitors in oesophagogastric cancer

**DOI:** 10.1038/s44276-023-00005-x

**Published:** 2023-08-02

**Authors:** Yohei Nose, Takuro Saito, Yukinori Kurokawa, Tsuyoshi Takahashi, Kazuyoshi Yamamoto, Kota Momose, Kotaro Yamashita, Koji Tanaka, Tomoki Makino, Hidetoshi Eguchi, Yuichiro Doki, Hisashi Wada

**Affiliations:** 1 Department of Gastroenterological Surgery, Graduate School of Medicine, Osaka University, Suita, Osaka Japan; 2 Department of Clinical Research in Tumor Immunology, Graduate School of Medicine, Osaka University, Suita, Osaka Japan

## Abstract

**Background:**

The treatment efficacy of immune checkpoint inhibitors (ICIs) is limited, and biomarkers that identify responders are urgently needed. We investigated whether C-reactive protein (CRP) kinetics are associated with the treatment efficacy of ICIs and prognosis in oesophagogastric cancers.

**Methods:**

We analysed 76 gastric cancer patients treated with nivolumab monotherapy. Patients were classified as CRP-spike, CRP-flat or CRP-increase according to CRP kinetics within 6 weeks after nivolumab initiation, and the treatment response and prognosis were compared. We further validated this classification in 71 oesophageal cancer patients with nivolumab monotherapy.

**Results:**

In the gastric cancer cohort, the CRP-spike, CRP-flat, and CRP-increase subgroups included 9, 37 and 30 patients, respectively. The CRP-spike subgroup had higher disease control rates than the CRP-increase subgroup (*P* = 0.0068) and had significantly better progression-free survival (PFS) (vs. CRP-flat: *P* = 0.045, CRP-increase: *P* = 0.0001). Multivariate analysis for PFS identified CRP-spike (HR = 0.38, *P* = 0.029) as an independent favourable prognostic factor. In the oesophageal cancer cohort, the CRP-spike, CRP-flat, and CRP-increase subgroups included 13, 27 and 31 patients, respectively, and multivariate analysis for PFS also identified CRP-spike (HR = 0.28, *P* = 0.0044) as an independent favourable prognostic factor.

**Conclusions:**

CRP kinetics may be useful in predicting the long-term outcome of nivolumab treatment in oesophagogastric cancers.

## Introduction

Immune checkpoint inhibitors (ICIs) have demonstrated anti-tumour responses in cancer patients and are currently approved for various types of cancer, including oesophagogastric cancers [1–3]. Anti-programmed cell death (PD)-1 monotherapy was used in Japan several years ago as third-line or later treatment of gastric cancer and as second-line or later treatment of oesophageal cancer [4, 5]. Currently, the combination of anti-PD-1 treatment and chemotherapy is the first-line treatment for recurrent or unresectable gastric or oesophageal cancer, and the importance of ICIs has been increasing in the treatment of oesophagogastric cancers [6–11]. Although a significant number of patients benefit from ICIs, the clinical efficacy is limited to a small percentage of patients. Therefore, extensive efforts are underway to identify biomarkers that can predict treatment responses and prognosis [12–16].

The acute phase reactant, C-reactive protein (CRP), is widely used as a clinical marker of systemic inflammation. In terms of the prognostic relevance of CRP, high serum CRP levels before surgery correlate with poor prognosis in various types of cancer [17–19]. Similarly, in ICI treatment, high CRP levels before treatment are associated with poor prognosis in cancer patients [20–24], whereas elevated CRP levels within 1 week after ICI initiation predict favourable treatment efficacy and prognosis in patients with non-small cell lung cancer [25]. Thus, high serum CRP levels at baseline correlate with poor prognosis, whereas increased CRP levels after ICI initiation correlate with better prognosis. Therefore, the combination of serum CRP kinetics before and after ICI initiation could be used as a biomarker for predicting the treatment response and prognosis. As anti-PD-1 monotherapy is administered every 2 weeks and imaging performed every 6–8 weeks, we attempted to establish criteria to predict the treatment response and prognosis by CRP kinetics within 6 weeks after ICI initiation, before imaging is performed.

Here, we aimed to investigate whether a new definition of CRP kinetics after ICI initiation is associated with a better treatment response and prognosis in two independent cohorts of patients with oesophagogastric cancer.

## Methods

### Study design and patients

We retrospectively analysed 76 patients with histologically confirmed gastric adenocarcinoma who were refractory to standard therapy and underwent anti-PD-1 treatment (nivolumab) alone between 2017 and 2022 at Osaka University Hospital (Osaka, Japan) as the discovery cohort. They had received at least two or more lines of systemic chemotherapy and had no previous ICI treatment. We further analysed 71 patients with histologically confirmed oesophageal squamous cell carcinoma treated with nivolumab alone between 2014 and 2022 at Osaka University Hospital as the validation cohort. They had received at least one line of systemic chemotherapy and had no previous ICI treatment. Our new definition of CRP kinetics was tested to predict treatment efficacy and prognosis in the two cohorts. A physical examination and laboratory tests were performed at baseline, before the initiation of nivolumab, and then at least every 2 weeks during nivolumab treatment. The patients received 3 mg/kg nivolumab every 2 weeks in 6-week cycles, but the intervals depended on the patient’s condition. Treatment was continued until disease progression, death, unacceptable toxic effects, or a patient’s request to discontinue. Those who died by 6 weeks after nivolumab administration were not included in either cohort. All patients provided written informed consent according to the guidelines of the Declaration of Helsinki. The study was approved by the Institutional Ethics Committee of Osaka University Hospital (Osaka, Japan, approval number 08226).

### Serum lab values

Serum CRP levels were analysed at baseline and weeks 1, 2, 4 and 6 after nivolumab initiation, as well as baseline serum levels of haemoglobin, lactate dehydrogenase (LDH), and albumin and the neutrophil-to-lymphocyte ratio (NLR). Based on previous reports, the cut-off for baseline CRP levels was set at 1.0 mg/dL [20, 24, 26]. Other measures were categorised into two groups based on the median value as the cut-off for statistical analysis.

### Definition of CRP kinetics

Patients were divided into the following three groups based on their CRP kinetics within 6 weeks after nivolumab initiation: CRP-spike, CRP-flat and CRP-increase. “CRP-spike” was defined as an increase in the CRP level more than twofold from baseline and >1.0 mg/dL within 4 weeks after nivolumab initiation, followed by a decrease of <1.0 mg/dL within 6 weeks. “CRP-flat” was defined as maintenance of the CRP level at <1.0 mg/dL or decreased from being >1.0 mg/dL to being <1.0 mg/dL during the entire period from before to 6 weeks after nivolumab initiation. All other patients were classified as “CRP-increase”.

### Assessment of treatment efficacy

Tumour responses were assessed by computed tomography (CT) approximately every 6 weeks and defined according to the Response Evaluation Criteria in Solid Tumours (RECIST), version 1.1 [27], based on the results of CT examinations using the following categories: complete response (CR), partial response (PR), stable disease (SD) and progressive disease (PD). The best overall response was determined based on the results of two CT scans from the start of nivolumab treatment. Patients who could not undergo the first CT for the evaluation of treatment efficacy due to disease progression or death were judged as having PD. Disease control rate (DCR) was defined as the percentage of patients with CR, PR and SD. The overall response rate (ORR) was defined as the percentage of patients with CR and PR. Progression-free survival (PFS) was defined as the time from nivolumab initiation to either disease progression or death from any cause. Overall survival (OS) was defined as the time from nivolumab initiation to death from any cause.

### Statistical analysis

Differences between the two or three groups were analysed using Fisher’s exact test, Pearson’s chi-squared, or the Kruskal–Wallis test as appropriate. Tumour responses among the groups were compared using Fisher’s exact test. Survival curves were calculated using the Kaplan–Meier method, and differences were assessed using the Log-rank test. Univariate and multivariate Cox proportional hazards regression analyses were carried out concerning PFS and OS after nivolumab initiation. Variables with *P* < 0.1 in the univariate analysis were included in the multivariate model. Some missing data for histology and HER2 status were excluded from the analysis. A value of *P* <  0.05 was considered significant. All statistical analyses were performed using JMP Pro 14 Discovery™ (SAS Institute Inc., Cary, NC, USA).

## Results

### Patient characteristics

The baseline characteristics of patients with gastric cancer are summarised in Table 1a. The median age was 68.5 years, 59 patients (77.6%) were male, and 55 patients (72.4%) had an Eastern Cooperative Oncology Group (ECOG) performance status (PS) score ≥1. Gastrectomy was performed in 44 patients (57.9%).

**Table 1:** Patient characteristics.

(a) Gastric cancer						
		Overall (*n* = 76)	CRP-spike (*n* = 9)	CRP-flat (*n* = 37)	CRP-increase (*n* = 30)	*P* value
Age (years)	Median	68.5	66	69	68.5	0.67
Sex	Male/female	59/17	8/1	30/7	21/9	0.46
ECOG-PS	0/1–2	21/55	4/5	15/22	2/28	**0.0020**
Histology	Differentiated/undifferentiated/unknown	31/39/6	4/5/0	17/17/3	10/17/3	0.63
HER2 status	Positive/negative/unknown	17/48/11	1/7/1	9/23/5	7/18/5	0.85
Liver metastasis	Positive/negative	35/41	5/4	18/19	12/18	0.69
Number of metastatic organs	≤1/ ≥ 2	43/33	6/3	22/15	15/15	0.65
Previous surgery	Presence/absence	44/32	6/3	25/12	13/17	0.13
LDH (U/L)	Median	214	193	194	226	0.063
Haemoglobin (g/dL)	Median	10.6	9.4	10.8	10.3	0.084
NLR	Median	2.37	1.62	2.27	3.28	**0.0074**
Albumin (g/dL)	Median	3.50	3.50	3.60	3.30	**0.024**
CRP (mg/dL)	Median	0.36	0.53	0.08	0.71	**<0.0001**
**(b) Oesophageal cancer**						
		**Overall (*n*** = 71)	**CRP-spike (*n*** = 13)	**CRP-flat (*n*** = 27)	**CRP-increase (*n*** = 31)	***P*** **value**
Age (years)	Median	70	66	70	72	0.33
Sex	Male/female	57/14	10/3	22/5	25/6	0.94
ECOG-PS	0/1–2	56/15	11/2	24/3	21/10	0.15
Liver metastasis	Positive/negative	14/57	1/12	3/24	10/21	0.083
Number of metastatic organs	≤1/ ≥ 2	36/35	5/8	19/8	12/19	**0.037**
Previous surgery	Presence/absence	48/23	8/5	21/6	19/12	0.38
Previous radiation therapy	Presence/absence	45/26	9/4	15/12	21/10	0.62
LDH (U/L)	Median	175	186.5	173.5	174	0.79
Haemoglobin (g/dL)	Median	11.2	11.7	11.2	10.7	**0.019**
NLR	Median	3.65	3.73	2.56	4.42	**0.0063**
Albumin (g/dL)	Median	3.8	3.9	3.9	3.6	**0.0002**
CRP (mg/dL)	Median	0.45	0.3	0.13	1.41	**<0.0001**

*ECOG-PS* Eastern Cooperative Oncology Group-Performance Status, *HER2* Human epidermal growth factor receptor 2, *LDH* lactate dehydrogenase (baseline), *NLR* neutrophil-to-lymphocyte ratio (baseline), *CRP* C-reactive protein (baseline).

Factors with P-values less than 0.05 were shown in bold.

The baseline characteristics of patients with oesophageal cancer are summarised in Table 1b. The median age was 70 years, 57 patients (80.3%) were male, and 15 patients (21.1%) had PS scores ≥1. Radiation therapy and oesophagectomy had been performed in 45 (63.4%) and 48 patients (67.6%), respectively.

### CRP kinetics and clinical response in gastric cancer

**Fig. 1:**
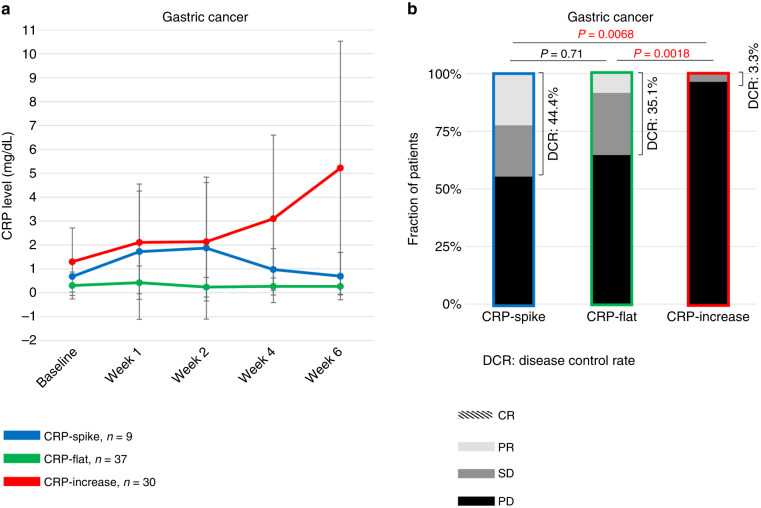
Changes in serum CRP levels after nivolumab administration among three CRP kinetics subgroups in gastric cancer. The graph shows the mean ± standard deviation for each group (**a**). Distribution of treatment responses according to RECIST among the three CRP kinetics subgroups in gastric cancer (**b**). *P* values were calculated using Fisher’s exact test for the disease control rate. CRP C-reactive protein, DCR disease control rate, CR complete response, PR partial response, SD stable disease, PD progressive disease.

The CRP kinetics before and after nivolumab treatment in the gastric cancer cohort is shown in Fig. 1a. The CRP-spike, CRP-flat and CRP-increase subgroups included 9 (11.8%), 37 (48.7%) and 30 (39.5%) patients, respectively. Patient characteristics in each group are summarised in Table 1a. Patients characterised as CRP-spike had better ECOG-PS (*P* = 0.018) and lower baseline NLR (*P* = 0.012) than those characterised as CRP-increase. Patients characterised as CRP-spike had significantly higher baseline CRP levels than those characterised as CRP-flat (*P* = 0.0024). There were no significant differences in the other background factors between patients characterised as CRP-spike and those with the other groups.

The best overall response was PR in 2 (22.2%), SD in 2 (22.2%), and PD in 5 (55.6%) in the CRP-spike subgroups; PR in 3 (8.1%), SD in 10 (27.0%), and PD in 24 (64.9%) in the CRP-flat subgroup; and PR in 0 (0%), SD in 1 (3.3%), and PD in 29 (96.7%) in the CRP-increase subgroup. The DCR was 44.4%, 35.1%, and 3.3% for the CRP-spike, CRP-flat, and CRP-increase subgroups, respectively, and the ORR was 22.2%, 8.1%, and 0%, respectively (Fig. 1b). Patients characterised as CRP-spike had significantly better DCR and ORR than those characterised as CRP-increase (*P* = 0.0068 and *P* = 0.049, respectively).

### CRP kinetics and survival in gastric cancer

**Fig. 2:**
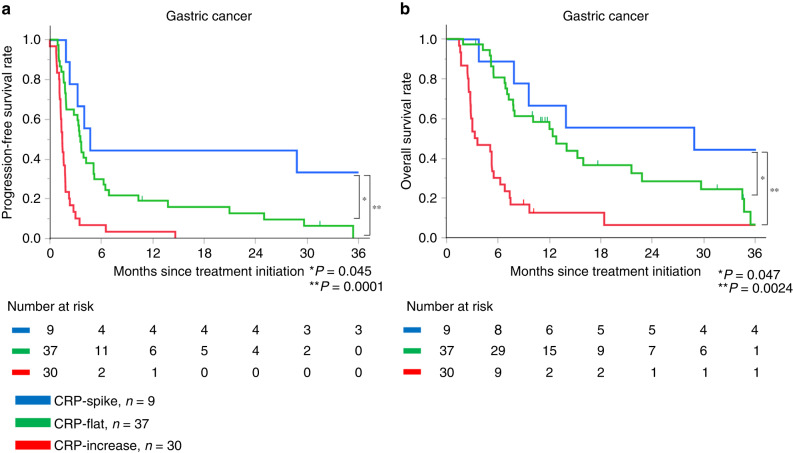
Survival analysis among three CRP kinetics subgroups in gastric cancer. Kaplan–Meier progression-free survival (**a**) and overall survival (**b**) after nivolumab initiation based on three CRP kinetics subgroups in gastric cancer.

The median follow-up was 7.6 months (range, 1.4–69.5 months). Median PFS was 4.7, 3.6 and 1.5 months, and median OS was 28.8, 12.8 and 3.4 months in patients characterised as CRP-spike, CRP-flat and CRP-increase, respectively. Patients characterised as CRP-spike had significantly better PFS (vs. CRP-flat, *P* = 0.045; vs. CRP-increase, *P* = 0.0001) and OS (vs. CRP-flat, *P* = 0.047; vs. CRP-increase, *P* = 0.0024) than the other subgroups (Fig. 2a, b).

We performed a univariate analysis with possible affecting factors for treatment efficacy from previous reports; patient’s backgrounds including PS, pathological factors (histology, HER2 status), treatment history (previous surgery, radiation therapy), and pre-treatment blood markers (LDH, Haemoglobin, NLR, Albumin, CRP) as well as metastatic sites (liver metastasis, number of metastatic organs). In a univariate analysis for PFS, PS 0 (*P* = 0.0087), lower baseline NLR (*P* = 0.027) and CRP-spike (*P* = 0.0074) were the significant favourable prognostic factors (Table 2a). Multivariate analysis for PFS identified CRP-spike (hazard ratio [HR] 0.38, 95% confidence interval [CI] 0.16–0.91; *P* = 0.029) as the independent favourable prognostic factor, as well as PS 0 (HR 2.17, 95% CI 1.25–3.75; *P* = 0.0057) and lower baseline NLR (HR 1.80, 95% CI 1.11–2.92; *P* = 0.017). In a univariate analysis for OS, PS 0 (*P* = 0.011), lower NLR (*P* = 0.037), and CRP-spike (*P* = 0.018) were the significant favourable prognostic factors. In multivariate analysis for OS, lower NLR (HR 1.74, 95% CI 1.03–2.93; *P* = 0.039) was the independent favourable prognostic factor, and CRP-spike (HR 0.44, 95% CI 0.18–1.08; *P* = 0.072) showed a trend toward favourable prognosis (Supplemental Table 1a).

**Table 2:** Univariate and multivariate analysis for progression-free survival.

(a) Gastric cancer							
		Univariate			Multivariate		
		HR	95% CI	*P* value	HR	95% CI	*P* value
Age (years)	≥69/ < 69	1.08	0.68–1.71	0.75			
Sex	Male/female	0.77	0.44–1.33	0.35			
**ECOG-PS**	**1–2/0**	**2.03**	**1.20–3.45**	**0.0087**	**2.17**	**1.25–3.75**	**0.0057**
Histology	Differentiated/undifferentiated	1.21	0.74–1.97	0.45			
HER2 status	Positive/negative	1.50	0.85–2.65	0.16			
Liver metastasis	Positive/negative	0.86	0.54–1.38	0.52			
Number of metastatic organs	≥2/ ≤ 1	0.94	0.58–1.52	0.81			
Previous gastrectomy	Presence/absence	0.70	0.44–1.12	0.14			
LDH (U/L)	≥214/ < 214	1.32	0.82–2.13	0.26			
Haemoglobin (g/dL)	≥10.6/ < 10.6	0.99	0.62–1.57	0.96			
**NLR**	**≥2.37/** < **2.37**	**1.69**	**1.06–2.70**	**0.027**	**1.80**	**1.11–2.92**	**0.017**
Albumin (g/dL)	≥3.5/ < 3.5	0.72	0.45–1.14	0.16			
CRP (mg/dL)	≥1.0/ < 1.0	1.48	0.83–2.62	0.18			
**CRP kinetics**	**Spike/others**	**0.31**	**0.13–0.73**	**0.0074**	**0.38**	**0.16–0.91**	**0.029**
**(b) Oesophageal cancer**							
Age (years)	≥70/ < 70	1.09	0.65–1.83	0.74			
Sex	Male/female	1.27	0.65–2.47	0.49			
ECOG-PS	1–2/0	1.37	0.74–2.53	0.32			
Liver metastasis	Positive/negative	1.03	0.54–1.93	0.94			
Number of metastatic organs	≥2/ ≤ 1	0.93	0.56–1.55	0.79			
Previous surgery	Presence/absence	1.28	0.72–2.26	0.40			
Previous radiation therapy	Presence/absence	0.88	0.52–1.49	0.63			
LDH (U/L)	≥175/ < 175	0.87	0.50–1.51	0.62			
Haemoglobin (g/dL)	≥11.2/ < 11.2	0.77	0.46–1.27	0.30			
NLR	≥3.65/ < 3.65	1.16	0.70–1.94	0.56			
Albumin (g/dL)	≥3.8/ < 3.8	0.52	0.31–0.88	0.014	0.78	0.44–1.39	0.39
CRP (mg/dL)	≥1.0/ < 1.0	2.29	1.33–3.96	0.0029	1.83	0.99–3.38	0.054
**CRP kinetics**	**Spike/others**	**0.22**	**0.095–0.53**	**0.0006**	**0.28**	**0.12–0.68**	**0.0044**

*HR* hazard ratio, *CI* confidence interval, *ECOG-PS* Eastern Cooperative Oncology Group-Performance Status, *HER2* Human epidermal growth factor receptor 2, *LDH* lactate dehydrogenase (baseline), *NLR* neutrophil-to-lymphocyte ratio (baseline), *CRP* C-reactive protein (baseline).

Factors with P-values less than 0.05 in multivariate analysis are shown in bold.

### CRP kinetics and clinical response in oesophageal cancer

**Fig. 3:**
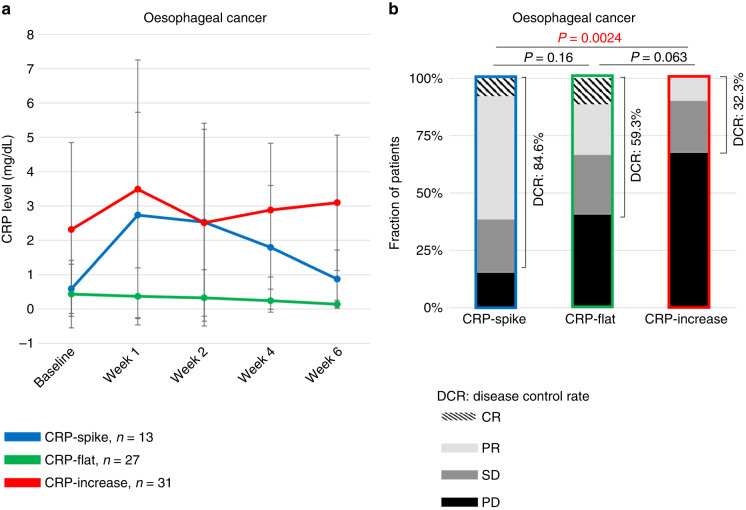
Changes in serum CRP levels after nivolumab administration among three CRP kinetics subgroups in oesophageal cancer. The graph shows the mean ± standard deviation for each group (**a**). Distribution of treatment responses according to RECIST among the three CRP kinetics subgroups in oesophageal cancer (**b**). *P* values were calculated using Fisher’s exact test for the disease control rate. CRP C-reactive protein, DCR disease control rate, CR complete response, PR partial response, SD stable disease, PD progressive disease.

The CRP kinetics before and after nivolumab treatment in the oesophageal cancer cohort is shown in Fig. 3a. The CRP-spike, CRP-flat and CRP-increase subgroups included 13 (18.3%), 27 (38.0%) and 31 (43.7%) patients, respectively. Patient characteristics in each group are summarised in Table 1b. There were significant differences in the number of metastatic organs and haematological factors, such as baseline haemoglobin, NLR, albumin, and CRP, among the three groups.

The best overall response was CR in 1 (7.7%), PR in 7 (53.8%), SD in 3 (23.1%) and PD in 2 (15.4%) in the CRP-spike subgroup; CR in 3 (11.1%), PR in 6 (22.2%), SD in 7 (25.9%) and PD in 11 (40.7%) in the CRP-flat subgroup; and CR in 0 (0%), PR in 3 (9.7%), SD in 7 (22.6%) and PD in 21 (67.7%) in the CRP-increase subgroup. The DCR was 84.6%, 59.3% and 32.3% for the patients in the CRP-spike, CRP-flat, and CRP-increase subgroups, respectively, and the ORR was 61.5%, 33.3%, and 9.7%, respectively. Patients characterised as CRP-spike had significantly better DCR and ORR than those characterised as CRP-increase (*P* = 0.0024 and *P* = 0.0008, respectively). We found no significant differences in DCR or ORR between patients characterised as CRP-spike and CRP-flat (Fig. 3b).

### CRP kinetics and survival in oesophageal cancer

**Fig. 4:**
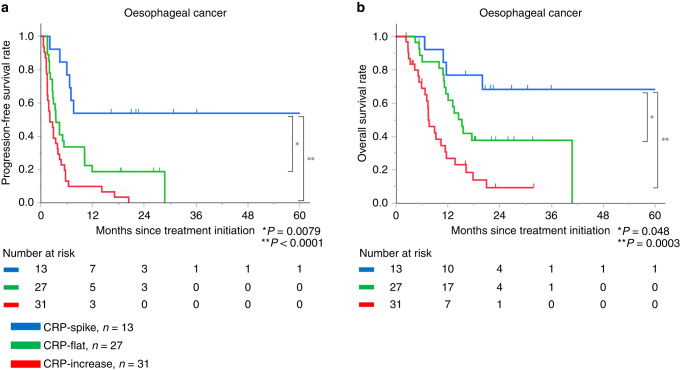
Survival analysis among three CRP kinetics subgroups in oesophageal cancer. Kaplan–Meier progression-free survival (**a**) and overall survival (**b**) after nivolumab initiation based on three CRP kinetics subgroups in oesophageal cancer.

The median follow-up was 11.6 months (range, 2.3–103.7 months). Median PFS was 98.6, 3.4 and 2.0 months and median OS not-yet-reached, 15.3 and 7.6 months for patients characterised as CRP-spike, CRP-flat, and CRP-increase, respectively. Patients characterised as CRP-spike had significantly better PFS (vs. CRP-flat, *P* = 0.0079; vs. CRP-increase, *P* < 0.0001) and OS (vs. CRP-flat, *P* = 0.048; vs. CRP-increase, *P* = 0.0003) than the other subgroups (Fig. 4a, b).

In a univariate analysis for PFS, higher baseline albumin (*P* = 0.014), lower baseline CRP (*P* = 0.0029), and CRP-spike (*P* = 0.0006) were the significant favourable prognostic factors (Table 2b). Multivariate analysis for PFS identified CRP-spike (HR 0.28, 95% CI 0.12–0.68; *P* = 0.0044) as the only independent favourable prognostic factor. In a univariate analysis for OS, higher baseline albumin (*P* = 0.0065), lower baseline CRP (*P* = 0.0001) and CRP-spike (*P* = 0.007) were the significant favourable prognostic factors. Multivariate analysis for OS also identified CRP-spike (HR 0.33, 95% CI 0.11–0.97; *P* = 0.043) as the independent favourable prognostic factor, as well as low baseline CRP (HR 2.38, 95% CI 1.14–4.96; *P* = 0.021; Supplemental Table 1b).

## Discussion

In this study, we identified and validated new CRP kinetics criteria within 6 weeks after ICI initiation that predicts treatment response to ICIs and prognosis in oesophagogastric cancers. We found that patients characterised as CRP-spike had better treatment responses and prognosis than the other groups. Therefore, we propose that the evaluation of early CRP kinetics after ICI initiation should be used as a predictive biomarker of treatment efficacy in patients with oesophagogastric cancers treated by ICIs.

As the treatment efficacy of ICIs in gastric and oesophageal cancer is limited, biomarkers for ICI treatment have been investigated intensively, including PD-L1 expression, microsatellite instability, tumour mutation burden, and Epstein-Barr virus infection [28]. Tumour PD-L1 expression is clinically used as a biomarker in ICI treatment of some types of cancer [29], but the predictive value of PD-L1 expression remains controversial in gastric cancer [4, 30–33]. Furthermore, tumour biomarkers are evaluated using tumour samples, which can be difficult to collect, especially in recurrent disease, so blood biomarkers may be useful in terms of sample collection and frequency of collection. To date, several studies have shown that inflammatory blood biomarkers predict the treatment efficacy of ICIs [25, 34, 35]. In particular, high CRP levels before ICI treatment are associated with poor treatment response and prognosis [20–24]; conversely, elevated CRP levels within 1 week after ICI initiation predict good treatment efficacy and prognosis [25]. Furthermore, recent reports have shown that a CRP flare response, defined as a transient increase in serum CRP levels after ICI initiation followed by a subsequent decrease below baseline, is associated with better prognosis in renal cell carcinoma, urothelial carcinoma, and lung cancer [26, 34, 36–38]. However, the definition was calculated with CRP kinetics within 12 weeks after ICI initiation, which is slower than the first imaging to examine clinical efficacy. To predict the treatment response and prognosis earlier than the first imaging evaluations, we devised a new definition of CRP kinetics within 6 weeks after ICI initiation. The definition predicted the treatment response and prognosis in two types of cancers. As CRP levels can be measured easily using clinical blood tests, this new definition of CRP kinetics may be a useful clinical tool for predicting the treatment response to ICIs.

Although we demonstrated that patients characterised as CRP-spike had a better treatment response and prognosis, it is unclear what the CRP elevation and subsequent decrease reflect. In general, a CRP elevation reflects systemic inflammation caused by infection or tumour [39–42]. However, transient CRP elevation early after ICI administration may be caused by the activation of anti-tumour immunity by the ICI treatment [34]. An inflammatory cytokine, IL-6, is released by activated dendritic cells, macrophages, and lymphocytes after ICI administration, and serum IL-6 elevation early after ICI administration is associated with a favourable prognosis [25, 35, 43]. As CRP production is stimulated by IL-6, an increase in CRP levels early after ICI administration may reflect the activation of immune cells, which can enhance anti-tumour immunity. However, a subsequent decrease in CRP levels is considered to represent tumour shrinkage because CRP levels have been reported to be proportional to tumour size [44]. The other explanation for the subsequent decrease in CRP levels was an improved immune status in the tumour microenvironment [36]. On the other hand, patients characterised as CRP-increase had late CRP elevation, which may represent a tumour-derived exacerbation of inflammation rather than activation of anti-tumour immunity. Overall, CRP-spike may reflect the early post-treatment activation of anti-tumour immunity and subsequent tumour shrinkage. The detailed mechanism underlying CRP-spike will require further investigation in the future.

In addition to CRP, age, PS, LDH, NLR, and albumin have been reported to correlate with the treatment efficacy and prognosis of ICI treatment [22, 34, 37, 45–48]. The present study showed that CRP-increase is associated with higher PS, LDH, and NLR and lower haemoglobin and albumin in the gastric cancer cohort, and higher NLR and lower haemoglobin and albumin in the oesophageal cancer cohort. In contrast, CRP-spike was associated with lower PS and NLR in the gastric cancer cohort and higher haemoglobin and albumin in the oesophageal cancer cohort. Considering these background factors, CRP kinetics may reflect the baseline host immunological status before ICI treatment. Thus, patients characterised as CRP-spike may have a better immunological status before ICI treatment than those characterised as CRP-flat or CRP-increase. The ORR of nivolumab monotherapy was reported to be 11.2% in gastric cancer and 17.2% in oesophageal cancer, which corresponds to 11.8% and 18.3% of patients characterised as CRP-spike in this study. Therefore, our definition of CRP kinetics successfully detected responders to ICI treatment [4, 5].

The present study had several limitations. First, it was a retrospective study with a relatively small sample size at a single institution. Therefore, no conclusions can be made. Second, data from blood tests and histology, including HER2 status, was deficient for some patients due to the retrospective nature of the study. The utility of the new definition of CRP kinetics needs to be validated in a large-scale prospective trial.

In conclusion, CRP kinetics could be useful for predicting the prognosis of ICI treatment in patients with gastric and oesophageal cancer. Further studies with a larger number of patients and various cancer types are needed to validate the new CRP kinetics criteria as a useful biomarker in ICI treatment.

## Supplementary information

Supplementary Table 1

## Data Availability

The datasets generated and/or analysed during the current study are available from the corresponding author on reasonable request.

## References

[1] Ando K, Hu Q, Kasagi Y, Oki E, Mori M. Recent developments in cancer research: expectations for a new remedy. Ann Gastroenterol Surg. 2021;5:419–26. 34337290 10.1002/ags3.12440 PMC8316733

[2] Miyamoto Y, Ogawa K, Ohuchi M, Tokunaga R, Baba H. Emerging evidence of immunotherapy for colorectal cancer. Ann Gastroenterol Surg. 2023;7:216–24. 36998297 10.1002/ags3.12633 PMC10043776

[3] Narita Y, Muro K. Updated immunotherapy for gastric cancer. J Clin Med. 2023;12:2636. 37048719 10.3390/jcm12072636 PMC10094960

[4] Kang YK, Boku N, Satoh T, Ryu MH, Chao Y, Kato K, et al. Nivolumab in patients with advanced gastric or gastro-oesophageal junction cancer refractory to, or intolerant of, at least two previous chemotherapy regimens (ONO-4538-12, ATTRACTION-2): a randomised, double-blind, placebo-controlled, phase 3 trial. Lancet. 2017;390:2461–71. 28993052 10.1016/S0140-6736(17)31827-5

[5] Satoh T, Kato K, Ura T, Hamamoto Y, Kojima T, Tsushima T, et al. Five-year follow-up of nivolumab treatment in Japanese patients with esophageal squamous-cell carcinoma (ATTRACTION-1/ONO-4538-07). Esophagus. 2021;18:835–43. 33993388 10.1007/s10388-021-00850-0 PMC8387268

[6] Kang YK, Chen LT, Ryu MH, Oh DY, Oh SC, Chung HC, et al. Nivolumab plus chemotherapy versus placebo plus chemotherapy in patients with HER2-negative, untreated, unresectable advanced or recurrent gastric or gastro-oesophageal junction cancer (ATTRACTION-4): a randomised, multicentre, double-blind, placebo-controlled, phase 3 trial. Lancet Oncol. 2022;23:234–47. 35030335 10.1016/S1470-2045(21)00692-6

[7] Janjigian YY, Shitara K, Moehler M, Garrido M, Salman P, Shen L, et al. First-line nivolumab plus chemotherapy versus chemotherapy alone for advanced gastric, gastro-oesophageal junction, and oesophageal adenocarcinoma (CheckMate 649): a randomised, open-label, phase 3 trial. Lancet. 2021;398:27–40. 34102137 10.1016/S0140-6736(21)00797-2 PMC8436782

[8] Doki Y, Ajani JA, Kato K, Xu J, Wyrwicz L, Motoyama S, et al. Nivolumab combination therapy in advanced esophageal squamous-cell carcinoma. N Engl J Med. 2022;386:449–62. 35108470 10.1056/NEJMoa2111380

[9] Sun JM, Shen L, Shah MA, Enzinger P, Adenis A, Doi T, et al. Pembrolizumab plus chemotherapy versus chemotherapy alone for first-line treatment of advanced oesophageal cancer (KEYNOTE-590): a randomised, placebo-controlled, phase 3 study. Lancet. 2021;398:759–71. 34454674 10.1016/S0140-6736(21)01234-4

[10] Kato K, Cho BC, Takahashi M, Okada M, Lin CY, Chin K, et al. Nivolumab versus chemotherapy in patients with advanced oesophageal squamous cell carcinoma refractory or intolerant to previous chemotherapy (ATTRACTION-3): a multicentre, randomised, open-label, phase 3 trial. Lancet Oncol. 2019;20:1506–17. 31582355 10.1016/S1470-2045(19)30626-6

[11] Shitara K, Ajani JA, Moehler M, Garrido M, Gallardo C, Shen L, et al. Nivolumab plus chemotherapy or ipilimumab in gastro-oesophageal cancer. Nature. 2022;603:942–8. 35322232 10.1038/s41586-022-04508-4 PMC8967713

[12] Topalian SL, Taube JM, Anders RA, Pardoll DM. Mechanism-driven biomarkers to guide immune checkpoint blockade in cancer therapy. Nat Rev Cancer. 2016;16:275–87. 27079802 10.1038/nrc.2016.36 PMC5381938

[13] Taube JM, Klein A, Brahmer JR, Xu H, Pan X, Kim JH, et al. Association of PD-1, PD-1 ligands, and other features of the tumor immune microenvironment with response to anti-PD-1 therapy. Clin Cancer Res. 2014;20:5064–74. 24714771 10.1158/1078-0432.CCR-13-3271 PMC4185001

[14] Nose Y, Saito T, Yamamoto K, Yamashita K, Tanaka K, Yamamoto K, et al. The tissue-resident marker CD103 on peripheral blood T cells predicts responses to anti-PD-1 therapy in gastric cancer. Cancer Immunol Immunother. 2022;72:169–81. 10.1007/s00262-022-03240-2 PMC10991138 35776160

[15] Kamphorst AO, Pillai RN, Yang S, Nasti TH, Akondy RS, Wieland A, et al. Proliferation of PD-1+ CD8 T cells in peripheral blood after PD-1-targeted therapy in lung cancer patients. Proc Natl Acad Sci USA. 2017;114:4993–8. 28446615 10.1073/pnas.1705327114 PMC5441721

[16] Zou W, Wolchok JD, Chen L. PD-L1 (B7-H1) and PD-1 pathway blockade for cancer therapy: Mechanisms, response biomarkers, and combinations. Sci Transl Med. 2016;8:328rv4. 26936508 10.1126/scitranslmed.aad7118 PMC4859220

[17] Wong VK, Malik HZ, Hamady ZZ, Al-Mukhtar A, Gomez D, Prasad KR, et al. C-reactive protein as a predictor of prognosis following curative resection for colorectal liver metastases. Br J Cancer. 2007;96:222–5. 17211465 10.1038/sj.bjc.6603558 PMC2360008

[18] Nozoe T, Iguchi T, Adachi E, Matsukuma A, Ezaki T. Preoperative elevation of serum C-reactive protein as an independent prognostic indicator for gastric cancer. Surg Today. 2011;41:510–3. 21431483 10.1007/s00595-009-4297-x

[19] Lee JG, Cho BC, Bae MK, Lee CY, Park IK, Kim DJ, et al. Preoperative C-reactive protein levels are associated with tumor size and lymphovascular invasion in resected non-small cell lung cancer. Lung Cancer. 2009;63:106–10. 18513823 10.1016/j.lungcan.2008.04.011

[20] Hagi T, Kurokawa Y, Kawabata R, Omori T, Matsuyama J, Fujitani K, et al. Multicentre biomarker cohort study on the efficacy of nivolumab treatment for gastric cancer. Br J Cancer. 2020;123:965–72. 32616848 10.1038/s41416-020-0975-7 PMC7492241

[21] Tanaka K, Tanabe H, Sato H, Ishikawa C, Goto M, Yanagida N, et al. Prognostic factors to predict the survival in patients with advanced gastric cancer who receive later-line nivolumab monotherapy—The Asahikawa Gastric Cancer Cohort Study (AGCC). Cancer Med. 2022;11:406–16. 34845844 10.1002/cam4.4461 PMC8729046

[22] Sato S, Oshima Y, Matsumoto Y, Seto Y, Yamashita H, Hayano K, et al. The new prognostic score for unresectable or recurrent gastric cancer treated with nivolumab: a multi-institutional cohort study. Ann Gastroenterol Surg. 2021;5:794–803. 34755011 10.1002/ags3.12489 PMC8560603

[23] Yoshida T, Ichikawa J, Giuroiu I, Laino AS, Hao Y, Krogsgaard M, et al. C reactive protein impairs adaptive immunity in immune cells of patients with melanoma. J Immunother Cancer. 2020;8:e000234. 10.1136/jitc-2019-000234 PMC7204799 32303612

[24] Han CL, Meng GX, Ding ZN, Dong ZR, Chen ZQ, Hong JG, et al. The predictive potential of the baseline C-reactive protein levels for the efficiency of immune checkpoint inhibitors in cancer patients: a systematic review and meta-analysis. Front Immunol. 2022;13:827788. 35211122 10.3389/fimmu.2022.827788 PMC8861087

[25] Ozawa Y, Amano Y, Kanata K, Hasegwa H, Matsui T, Kakutani T, et al. Impact of early inflammatory cytokine elevation after commencement of PD-1 inhibitors to predict efficacy in patients with non-small cell lung cancer. Med Oncol. 2019;36:33. 30825015 10.1007/s12032-019-1255-3

[26] Fukuda S, Saito K, Yasuda Y, Kijima T, Yoshida S, Yokoyama M, et al. Impact of C-reactive protein flare-response on oncological outcomes in patients with metastatic renal cell carcinoma treated with nivolumab. J Immunother Cancer. 2021;9:e001564. 10.1136/jitc-2020-001564 PMC7896625 33602695

[27] Eisenhauer EA, Therasse P, Bogaerts J, Schwartz LH, Sargent D, Ford R, et al. New response evaluation criteria in solid tumours: revised RECIST guideline (version 1.1). Eur J Cancer. 2009;45:228–47. 19097774 10.1016/j.ejca.2008.10.026

[28] Kawazoe A, Shitara K, Boku N, Yoshikawa T, Terashima M. Current status of immunotherapy for advanced gastric cancer. Jpn J Clin Oncol. 2021;51:20–7. 33241322 10.1093/jjco/hyaa202

[29] Doroshow DB, Bhalla S, Beasley MB, Sholl LM, Kerr KM, Gnjatic S, et al. PD-L1 as a biomarker of response to immune-checkpoint inhibitors. Nat Rev Clin Oncol. 2021;18:345–62. 33580222 10.1038/s41571-021-00473-5

[30] Yamashita K, Iwatsuki M, Ajani JA, Baba H. Programmed death ligand-1 expression in gastrointestinal cancer: clinical significance and future challenges. Ann Gastroenterol Surg. 2020;4:369–78. 32724880 10.1002/ags3.12348 PMC7382440

[31] Fuchs CS, Doi T, Jang RW, Muro K, Satoh T, Machado M, et al. Safety and efficacy of pembrolizumab monotherapy in patients with previously treated advanced gastric and gastroesophageal junction cancer: phase 2 clinical KEYNOTE-059 trial. JAMA Oncol. 2018;4:e180013. 29543932 10.1001/jamaoncol.2018.0013 PMC5885175

[32] Shitara K, Özgüroğlu M, Bang YJ, Di Bartolomeo M, Mandalà M, Ryu MH, et al. Pembrolizumab versus paclitaxel for previously treated, advanced gastric or gastro-oesophageal junction cancer (KEYNOTE-061): a randomised, open-label, controlled, phase 3 trial. Lancet. 2018;392:123–33. 29880231 10.1016/S0140-6736(18)31257-1

[33] Shitara K, Van Cutsem E, Bang YJ, Fuchs C, Wyrwicz L, Lee KW, et al. Efficacy and safety of pembrolizumab or pembrolizumab plus chemotherapy vs chemotherapy alone for patients with first-line, advanced gastric cancer: the KEYNOTE-062 phase 3 randomized clinical trial. JAMA Oncol. 2020;6:1571–80. 32880601 10.1001/jamaoncol.2020.3370 PMC7489405

[34] Klümper N, Saal J, Berner F, Lichtensteiger C, Wyss N, Heine A, et al. C reactive protein flare predicts response to checkpoint inhibitor treatment in non-small cell lung cancer. J Immunother Cancer. 2022;10:e004024. 10.1136/jitc-2021-004024 PMC8928397 35292517

[35] Lim JU, Yoon HK. Potential predictive value of change in inflammatory cytokines levels subsequent to initiation of immune checkpoint inhibitor in patients with advanced non-small cell lung cancer. Cytokine. 2021;138:155363. 33264749 10.1016/j.cyto.2020.155363

[36] Tomisaki I, Harada M, Tokutsu K, Minato A, Nagata Y, Kimuro R, et al. Impact of C-reactive protein flare response in patients with advanced urothelial carcinoma who received pembrolizumab. In Vivo. 2021;35:3563–8. 34697195 10.21873/invivo.12659 PMC8627767

[37] Klümper N, Schmucker P, Hahn O, Höh B, Mattigk A, Banek S, et al. C-reactive protein flare-response predicts long-term efficacy to first-line anti-PD-1-based combination therapy in metastatic renal cell carcinoma. Clin Transl Immunology. 2021;10:e1358. 34925829 10.1002/cti2.1358 PMC8648498

[38] Klümper N, Sikic D, Saal J, Büttner T, Goldschmidt F, Jarczyk J, et al. C-reactive protein flare predicts response to anti-PD-(L)1 immune checkpoint blockade in metastatic urothelial carcinoma. Eur J Cancer. 2022;167:13–22. 35366569 10.1016/j.ejca.2022.02.022

[39] Saito K, Kihara K. C-reactive protein as a biomarker for urological cancers. Nat Rev Urol. 2011;8:659–66. 22025173 10.1038/nrurol.2011.145

[40] Sollie S, Michaud DS, Sarker D, Karagiannis SN, Josephs DH, Hammar N, et al. Chronic inflammation markers are associated with risk of pancreatic cancer in the Swedish AMORIS cohort study. BMC Cancer. 2019;19:858. 31464604 10.1186/s12885-019-6082-6 PMC6716919

[41] Wang D, DuBois RN. Immunosuppression associated with chronic inflammation in the tumor microenvironment. Carcinogenesis. 2015;36:1085–93. 26354776 10.1093/carcin/bgv123 PMC5006153

[42] Lukaszewicz-Zając M, Mroczko B, Gryko M, Kędra B, Szmitkowski M. Comparison between clinical significance of serum proinflammatory proteins (IL-6 and CRP) and classic tumor markers (CEA and CA 19-9) in gastric cancer. Clin Exp Med. 2011;11:89–96. 20938721 10.1007/s10238-010-0114-5 PMC3087107

[43] Hunter CA, Jones SA. IL-6 as a keystone cytokine in health and disease. Nat Immunol. 2015;16:448–57. 25898198 10.1038/ni.3153

[44] Kim DK, Oh SY, Kwon HC, Lee S, Kwon KA, Kim BG, et al. Clinical significances of preoperative serum interleukin-6 and C-reactive protein level in operable gastric cancer. BMC Cancer. 2009;9:155. 19457231 10.1186/1471-2407-9-155 PMC2694817

[45] De Giorgi U, Procopio G, Giannarelli D, Sabbatini R, Bearz A, Buti S, et al. Association of systemic inflammation index and body mass index with survival in patients with renal cell cancer treated with nivolumab. Clin Cancer Res. 2019;25:3839–46. 30967420 10.1158/1078-0432.CCR-18-3661

[46] Suzuki K, Terakawa T, Furukawa J, Harada K, Hinata N, Nakano Y, et al. C-reactive protein and the neutrophil-to-lymphocyte ratio are prognostic biomarkers in metastatic renal cell carcinoma patients treated with nivolumab. Int J Clin Oncol. 2020;25:135–44. 31512006 10.1007/s10147-019-01528-5

[47] Ishihara H, Tachibana H, Takagi T, Kondo T, Fukuda H, Yoshida K, et al. Predictive impact of peripheral blood markers and C-reactive protein in nivolumab therapy for metastatic renal cell carcinoma. Target Oncol. 2019;14:453–63. 31359231 10.1007/s11523-019-00660-6

[48] Ota Y, Takahari D, Suzuki T, Osumi H, Nakayama I, Oki A, et al. Changes in the neutrophil-to-lymphocyte ratio during nivolumab monotherapy are associated with gastric cancer survival. Cancer Chemother Pharmacol. 2020;85:265–72. 31907646 10.1007/s00280-019-04023-w

