# Supplementary material for: C-reactive protein kinetics as a predictive marker for long-term outcome of immune checkpoint inhibitors in oesophagogastric cancer
Source: BJC Rep. 2023 Aug 2;1:7. doi: 10.1038/s44276-023-00005-x (PMC11524005; doi:10.1038/s44276-023-00005-x)
Supplement: Supplementary file 1 — Supplementary Table 1 [file 44276_2023_5_MOESM1_ESM.docx]

Supplemental Table.1 Univariate and multivariate analysis for overall survival

| (a) Gastric cancer |  | | | | | | | |
| --- | --- | --- | --- | --- | --- | --- | --- | --- |
|  |  | univariate | | | multivariate | | | |
|  |  | HR* | 95%CI* | p value | HR | 95%CI | p value | |
| Age (years) | ≥ 69/< 69 | 1.02 | 0.62-1.69 | 0.93 |  |  |  | |
| Sex | Male/Female | 0.66 | 0.36-1.19 | 0.17 |  |  |  | |
| ECOG PS* | 1-2/0 | 2.18 | 1.19-3.98 | 0.011 | 1.96 | 1.00-3.84 | 0.051 | |
| Histology | Differentiated/Undifferentiated | 0.88 | 0.52-1.49 | 0.63 |  |  |  | |
| HER2* status | Positive/Negative | 0.73 | 0.39-1.36 | 0.32 |  |  |  | |
| Liver metastasis | Positive/Negative | 0.78 | 0.47-1.30 | 0.34 |  |  |  | |
| Number of metastatic organs | ≥2/≤1 | 0.92 | 0.55-1.53 | 0.75 |  |  |  | |
| Previous gastrectomy | Presence/Absence | 0.69 | 0.42-1.16 | 0.16 |  |  |  | |
| LDH* (U/L) | ≥ 214/< 214 | 1.56 | 0.92-2.64 | 0.10 |  |  |  | |
| Haemoglobin (g/dL) | ≥ 10.6/< 10.6 | 0.70 | 0.42-1.16 | 0.17 |  |  |  | |
| **NLR*** | **≥ 2.37/< 2.37** | **1.73** | **1.04-2.89** | **0.037** | **1.74** | **1.03-2.93** | **0.039** | |
| Albumin (g/dL) | ≥ 3.5/< 3.5 | 0.63 | 0.38-1.05 | 0.079 | 0.90 | 0.51-1.57 | 0.70 | |
| CRP* (mg/dL) | ≥ 1.0/< 1.0 | 1.23 | 0.65-2.33 | 0.52 |  |  |  | |
| CRP Kinetics | Spike/Others | 0.34 | 0.14-0.83 | 0.018 | 0.44 | 0.18-1.08 | 0.072 | |
|  | | | | | | | |  |
| (b) Esophageal cancer |  | | | | | | | |
|  |  | univariate | | | multivariate | | | |
|  |  | HR* | 95%CI* | p value | HR | 95%CI | p value | |
| Age (years) | ≥ 70/< 70 | 1.19 | 0.66-2.15 | 0.57 |  |  |  | |
| Sex | Male/Female | 1.38 | 0.61-3.10 | 0.44 |  |  |  | |
| ECOG PS* | 1-2/0 | 1.99 | 0.97-4.06 | 0.060 | 1.82 | 0.82-4.04 | 0.14 | |
| Liver metastasis | Positive/Negative | 1.59 | 0.78-3.24 | 0.20 |  |  |  | |
| Number of metastatic organs | ≥2/≤1 | 1.03 | 0.57-1.86 | 0.91 |  |  |  | |
| Previous surgery | Presence/Absence | 0.91 | 0.48-1.72 | 0.77 |  |  |  | |
| Previous radiation therapy | Presence/Absence | 0.84 | 0.46-1.52 | 0.56 |  |  |  | |
| LDH* (U/L) | ≥ 175/< 175 | 0.84 | 0.44-1.61 | 0.61 |  |  |  | |
| Haemoglobin (g/dL) | ≥ 11.2/< 11.2 | 0.78 | 0.44-1.41 | 0.42 |  |  |  | |
| NLR* | ≥ 3.65/< 3.65 | 1.56 | 0.86-2.83 | 0.14 |  |  |  | |
| Albumin (g/dL) | ≥ 3.8/< 3.8 | 0.43 | 0.23-0.79 | 0.0065 | 0.75 | 0.36-1.55 | 0.44 | |
| **CRP* (mg/dL)** | **≥ 1.0/< 1.0** | **3.28** | **1.79-6.00** | **0.0001** | **2.38** | **1.14-4.96** | **0.021** | |
| **CRP Kinetics** | **Spike/Others** | **0.24** | **0.086-0.68** | **0.007** | **0.33** | **0.11-0.97** | **0.043** | |

HR*: hazard ratio, CI*: Confidence interval, ECOG-PS*: Eastern Cooperative Oncology Group-Performance Status, HER2*: Human epidermal growth factor receptor 2, LDH*: lactate dehydrogenase (baseline), NLR*: neutrophil-to-lymphocyte ratio (baseline), CRP*: C-reactive protein (baseline)
